# Intra-Articular N-Acetylcysteine Reduces Synovitis Without Preventing Cartilage Degeneration in Experimental Osteoarthritis

**DOI:** 10.3390/biomedicines14010086

**Published:** 2025-12-31

**Authors:** Mustafa Dinç, Hünkar Çağdaş Bayrak, Recep Karasu, Bilal Aykaç, Ömer Cevdet Soydemir, Aysun Saricetin

**Affiliations:** 1 Orthopedics and Traumatology Clinics, Bursa City Hospital, Bursa 16250, Turkey; recepkarasu@hotmail.com (R.K.); draykac@gmail.com (B.A.); dromer77@hotmail.com (Ö.C.S.); 2 Orthopedics and Traumatology Clinics, Çekirge State Hospital, Bursa 16090, Turkey; cagdasbayrak90@gmail.com; 3 Department of Pathology, Faculty of Veterinary Medicine, Bursa Uludag University, Nilufer, Bursa 16059, Turkey; aysunsaricetin@gmail.com

**Keywords:** osteoarthritis, synovitis, cartilage degradation, N-acetylcysteine, intra-articular therapy

## Abstract

**Background/Objectives**: Osteoarthritis (OA) is a multifactorial degenerative joint disease characterized by synovial inflammation, oxidative stress, and progressive cartilage degeneration. This study investigated whether intra-articular N-acetylcysteine (NAC) attenuates synovial inflammation and oxidative stress and whether these effects translate into structural cartilage protection. **Methods**: OA was induced in rats by anterior cruciate ligament transection (ACLT). NAC (5 mg/50 µL) was administered intra-articularly once weekly for three weeks post-ACLT. Inflammatory cytokines (IL-1β, IL-6, TNF-α), oxidative stress markers (iNOS, TAS, TOS, OSI), and cartilage degradation markers (MMP-13, COMP, CTX-II) were quantified in synovial fluid and cartilage homogenates using ELISA. Cartilage integrity was evaluated histologically using the modified Mankin scoring system. **Results**: Compared with controls, NAC significantly reduced synovial IL-1β, IL-6, TNF-α, MMP-13, and iNOS levels and improved the synovial redox profile by increasing TAS and reducing TOS and OSI (all *p* < 0.05). In contrast, NAC did not significantly alter cartilage homogenate levels of inflammatory cytokines, oxidative stress indices, or degradation markers (COMP, CTX-II, MMP-13). Histological analysis demonstrated persistent cartilage fissuring, hypocellularity, and proteoglycan loss in both groups (*p* > 0.05). **Conclusions**: Intra-articular NAC exerts potent anti-inflammatory and antioxidative effects within the synovial compartment but fails to prevent cartilage degeneration in the ACLT model. These findings indicate a compartment-specific therapeutic profile, suggesting that NAC may function as a symptom-modifying agent in synovitis-dominant OA rather than a structure-modifying therapy. Future studies should focus on optimized delivery systems or combination strategies targeting cartilage and subchondral bone to achieve disease modification.

## 1. Introduction

Degenerative osteoarthritis (OA) is one of the most prevalent joint disorders in humans, particularly affecting the knee, and represents a leading cause of disability in the aging population [1,2,3,4,5,6]. Recent epidemiological and mechanistic reviews affirm that its prevalence continues to escalate with aging demographics and metabolic comorbidities [4,6,7]. Importantly, OA is now recognized as a whole-joint disease rather than an isolated cartilage disorder, involving the coordinated pathological alteration of multiple joint tissues, including articular cartilage, synovium, subchondral bone, ligaments, menisci, and the infrapatellar fat pad [8,9,10,11].

Pathophysiologically, OA arises from the interplay of mechanical overload and biochemical signaling. Degraded cartilage fragments trigger a synovial inflammatory response mediated by IL-1, IL-6, and TNF-α, which in turn induce catabolic enzymes such as MMPs to accelerate cartilage and periarticular tissue destruction [12,13,14]. Concurrently, oxidative stress driven by reactive oxygen and nitrogen species (ROS, NO) promotes chondrocyte apoptosis and matrix damage, feeding into a vicious cycle of degeneration [3,15,16,17].

In addition to soluble cytokines, cartilage degradation generates a wide spectrum of matrix-derived fragments, including fibronectin, aggrecan, and type II collagen peptides, which act as damage-associated molecular patterns (DAMPs) [18,19,20]. These fragments activate synovial macrophages and fibroblast-like synoviocytes through pattern-recognition receptors such as Toll-like receptors, thereby amplifying inflammatory signaling and matrix catabolism [21,22,23].

Reactive oxygen and nitrogen species in OA originate predominantly from activated synoviocytes, infiltrating inflammatory cells, and stressed chondrocytes via mitochondrial dysfunction, NADPH oxidase activity, and inducible nitric oxide synthase (iNOS) [24,25]. Excessive ROS/RNS production disrupts redox homeostasis, promotes lipid peroxidation and DNA damage, and further sensitizes cartilage tissue to inflammatory and mechanical injury [26,27,28].

Current management strategies, including oral medications, physiotherapy, and intra-articular interventions, primarily target pain and functional impairment but lack the capacity to modify structural disease progression [29,30,31,32]. This limitation highlights the need for disease-modifying approaches that can simultaneously alleviate inflammation, counteract oxidative stress, and preserve cartilage integrity.

Among the candidate agents explored for this purpose, N-acetylcysteine (NAC) is a well-established antioxidant with a long history of clinical use in respiratory and hepatic diseases [33,34]. Beyond musculoskeletal research, N-acetylcysteine has been extensively investigated in clinical and experimental settings for cardiovascular, neurodegenerative, renal, and oncological disorders, owing to its glutathione-replenishing capacity, direct free-radical scavenging properties, and modulation of redox-sensitive inflammatory pathways. Large-scale clinical trials and systematic reviews have demonstrated its safety and pleiotropic biological effects across multiple organ systems, supporting its translational relevance as a therapeutic antioxidant [35]. Experimental studies suggest that NAC may also modulate inflammatory and oxidative processes in OA. However, only a limited number of studies have directly investigated NAC in the context of OA, and their results have been inconsistent. Some reports indicate reductions in oxidative stress and matrix degradation, while others show limited or no cartilage-protective effects, particularly in chronic or mechanically induced disease models [36,37,38,39]. Moreover, an observational clinical study has raised the possibility that long-term NAC use could even be associated with increased OA risk [40].

Despite its common oral administration, systemic delivery of NAC is limited by first-pass metabolism, short plasma half-life, and insufficient joint bioavailability [41,42,43]. Intra-articular administration was therefore selected to achieve higher local concentrations within the synovial microenvironment, minimize systemic exposure, and directly target inflammation and oxidative stress at the site of pathology [32,44,45]. This localized approach is particularly relevant in OA, where synovial-driven inflammatory processes play a central role in symptom generation and disease propagation.

Given these conflicting data and the scarcity of comprehensive in vivo studies, there is a clear need to better define NAC’s role in OA. In this study, we aimed to clarify NAC’s compartment-specific actions in an anterior cruciate ligament transection (ACLT) rat model of knee OA. We adopted a compartmental analysis approach, evaluating biomarkers both in synovial fluid and cartilage homogenates (IL-1, IL-6, TNF-α, MMP-13, COMP, CTX-II, TAS, TOS) and correlated these with histological findings. This approach allowed us to test whether NAC’s anti-inflammatory and antioxidative effects in the synovial environment extend to structural cartilage protection, thereby defining its therapeutic potential and limitations in OA.

## 2. Materials and Methods

Twenty male Sprague–Dawley rats (12 weeks old, 250–300 g) were obtained from the Experimental Animals Breeding and Research Unit of Uludağ University Medical Faculty. Animals were housed under controlled laboratory conditions (22 ± 2 °C, 12 h light/dark cycle) with free access to standard chow and water. They were randomly allocated into two experimental groups (*n* = 10 per group) using a simple randomization method based on a random number generator to minimize selection bias. To further reduce bias, investigators responsible for sample collection, data acquisition, and analysis were blinded to group allocation, and all samples were numerically coded until the completion of statistical analysis [46,47].

Male rats were selected to provide a stable hormonal background and to avoid systemic confounders. Although ovariectomized (OVX) female rats are frequently used to mimic the postmenopausal state, this approach remains controversial. Some reports indicate that OVX induces cartilage degeneration resembling early osteoarthritis, whereas others describe only bone loss and osteoporosis-like changes without consistent joint pathology [48,49,50]. Furthermore, it has been suggested that OVX requires mature animals and long follow-up to produce measurable joint changes, limiting its utility in therapeutic intervention studies [51,52]. In addition, estrogen deficiency leads to systemic metabolic changes, including bone loss, weight gain, altered fat distribution, and increased inflammation, all of which may confound interpretation of treatment effects [48,53].

All procedures were approved by the Animal Welfare Committee of Uludağ University (Approval No: 2023-10/01; 26 September 2023) and carried out according to institutional and international ethical standards.

### 2.1. Surgical Induction of Osteoarthritis

A rat model of knee OA was established by transection of the anterior cruciate ligament (ACLT), a widely validated technique to induce chronic joint instability [54,55]. Anesthesia was induced with isoflurane (4% for induction, 2% for maintenance) delivered via inhalation [56]. Following aseptic preparation, a medial parapatellar incision was made to access the right knee. The ACL was exposed and transected with microsurgical scissors, and instability was confirmed using the anterior drawer test. The capsule was closed with 5–0 absorbable sutures and the skin with subcuticular stitches. No NSAIDs or opioids were given in order to avoid confounding anti-inflammatory effects. Animals were monitored daily for pain, distress, or infection, and resumed unrestricted cage activity after recovery, thereby mimicking physiological loading that promotes OA development.

From the first postoperative week, both groups received weekly intra-articular (IA) injections for 3 consecutive weeks. The control group was injected with sterile phosphate-buffered saline (PBS, 50 µL), whereas the NAC group received PBS containing 5 mg of NAC (5 mg/50 µL). NAC solutions were freshly prepared under sterile conditions immediately prior to injection. N-acetylcysteine (NAC) powder was obtained from Sigma-Aldrich (St. Louis, MO, USA; ≥99% purity, pharmaceutical grade) and dissolved in sterile phosphate-buffered saline (PBS) immediately prior to administration. Intra-articular injections were performed using sterile disposable 26-gauge needles (BD Microlance™, Becton Dickinson, Franklin Lakes, NJ, USA), which are commonly used for small-animal joint injections to minimize tissue trauma while ensuring accurate intra-articular delivery. Injections were performed under brief isoflurane anesthesia using a 26-gauge needle via the medial parapatellar approach, with free joint reflux confirming intra-articular placement. The intra-articular dose of 5 mg NAC was chosen based on previous in vivo studies demonstrating local efficacy and safety in ACLT and trauma-induced OA models [36,38]. This dose and weekly administration schedule were selected to specifically target the early post-traumatic inflammatory phase following anterior cruciate ligament transection, during which synovial activation and oxidative stress are most pronounced. Weekly dosing over three consecutive weeks was preferred to maintain local antioxidant exposure while minimizing repeated joint manipulation and potential anesthesia-related confounding effects [57,58]. Animals were sacrificed under deep anesthesia by decapitation 6 weeks after the first injection, a time point selected based on previous studies demonstrating that structural cartilage degeneration and subchondral bone remodeling reliably occur after anterior cruciate ligament transection. The ACLT model is characterized by progressive deterioration rather than spontaneous healing, and a minimum of 6 weeks is widely accepted to allow consistent OA pathology to develop [52,59,60,61,62,63]. Following euthanasia, joint tissues were harvested for analysis.

### 2.2. Histological Processing and Evaluation

Knee joint specimens, including the distal femur and proximal tibia, were excised en bloc and immediately fixed in 10% neutral-buffered formalin (containing 4% formaldehyde) for 48 h. Following fixation, samples were decalcified in 3% formic acid, with the solution replaced every 24 h, for 5–6 days, a protocol previously shown to provide adequate preservation of tissue morphology within a shorter time frame compared to ethylenediaminetetraacetic acid (EDTA) [64,65,66]. The endpoint of decalcification was verified by the ammonium oxalate precipitation test to ensure complete calcium removal [67]. After decalcification, tissues were processed through graded alcohols, cleared in xylene, and embedded in paraffin. Serial parasagittal sections of 5 µm thickness were obtained using a rotary microtome, ensuring representation from both medial and lateral compartments of the tibiofemoral joint. From each specimen, at least three non-consecutive sections were selected at 50–100 µm intervals to minimize sampling bias [68,69].

Hematoxylin–Eosin (H&E) staining: Sections were deparaffinized, rehydrated, and stained with Mayer’s hematoxylin (4–6 min), rinsed in tap water, blued with 0.2% ammonia water, and counterstained with eosin Y (30–60 s). Following dehydration in graded ethanols, clearing in xylene, and mounting, sections were examined for general tissue architecture and cellularity [70].

Safranin-O/Fast Green staining: Additional sections were stained with Weigert’s iron hematoxylin (5 min), washed, and counterstained with 0.02% Fast Green (2–3 min). After differentiation in 1% acetic acid (10–15 s), slides were stained with 0.1% Safranin-O (3–5 min) to visualize proteoglycan distribution. Sections were dehydrated, cleared in xylene, and coverslipped. Negative control slides were processed in parallel omitting the Safranin-O step [71].

Microscopic assessment was performed using an Olympus CX41 microscope (Olympus Corporation, Tokyo, Japan, magnifications 4×, 10×, 20×, 40×). Cartilage integrity, cellularity, surface regularity, and proteoglycan distribution were evaluated using the modified Mankin scoring system [72]. For Mankin scoring, evaluation was restricted to the medial tibiofemoral compartment, including the medial femoral condyle and medial tibial plateau, which are known to exhibit the earliest and most pronounced cartilage degeneration following anterior cruciate ligament transection. From each joint, two parasagittal sections obtained from comparable medial regions were analyzed, and the mean score was used for statistical evaluation. This region-specific approach was adopted to ensure anatomical consistency and to minimize variability related to compartment-dependent loading patterns inherent to the ACLT model. To minimize bias, two independent pathologists, blinded to group allocation, scored all sections. At least two sections from comparable anatomical regions of each joint were analyzed, and discrepancies were resolved by consensus.

In addition to cartilage assessment, synovial tissue was evaluated on the same sections. Hematoxylin–eosin–stained sections were examined for synovial lining thickness, stromal cellularity, inflammatory cell infiltration, and vascular congestion. Synovial evaluation was performed qualitatively by two independent observers blinded to group allocation, without the use of a predefined scoring system. 

#### 2.2.1. Retrieval and Handling of Synovial Fluid

Synovial fluid was collected after euthanasia using a perfusion technique adapted from Barton et al. [73]. Following insertion of two needles into the joint space (one for inflow and one for outflow), sterile PBS was infused at a constant rate of 100 µL/min. Perfusion was performed using a programmable syringe pump (Harvard Apparatus, Holliston, MA, USA) to ensure a constant and reproducible infusion rate. Sterile polyethylene tubing (PE-50; Becton Dickinson, Franklin Lakes, NJ, USA) was used for both inflow and outflow lines to maintain uniform flow dynamics and minimize dead volume during synovial fluid collection. After an initial 100 µL infusion to stabilize intra-articular pressure, perfusion continued until a 250 µL sample was collected from the outflow tubing, providing sufficient synovial fluid for biochemical analysis. This protocol ensured a uniform recovery volume across all animals and minimized variability related to dilution. The collected fluid was centrifuged at 3000× *g* for 10 min at 4 °C to remove cellular debris. Supernatants were carefully aspirated, divided into aliquots to avoid repeated freeze–thaw cycles, and stored at −80 °C until further analysis.

#### 2.2.2. Cartilage Tissue Extraction and Homogenization 

Articular cartilage was carefully dissected from the femoral condyles and tibial plateaus immediately after sacrifice, taking special care to avoid contamination from subchondral bone or meniscal tissue. The harvested cartilage was chopped into ~2 mm fragments, snap-frozen in liquid nitrogen, and pulverized in a pre-chilled porcelain mortar. The resulting powder was suspended in phosphate-buffered saline (PBS) at a ratio of 1:10 (*w*/*v*) and homogenized using an ultrasonic homogenizer (Ultra-Turrax T25, IKA, Staufen, Germany) equipped with a 2 mm diameter probe. Homogenization was performed at 70% amplitude in pulse mode (5 s on, 2 s off) for a total duration of 3 min, while samples were continuously maintained in an ice bath to prevent heat-induced protein degradation [74,75]. The homogenates were then centrifuged at 12,000× *g* for 20 min at 4 °C, and the supernatants were collected. Aliquots were stored at −80 °C until biochemical analysis. To minimize variability, all samples were processed under identical conditions, and protein concentrations were measured using a standard Bradford assay prior to ELISA-based cytokine and oxidative stress marker analysis [76].

#### 2.2.3. Quantification of Cytokines, Enzymes, and Degradation Markers by ELISA

Cytokine and biochemical marker concentrations in synovial fluid and cartilage homogenates were quantified using commercially available ELISA kits (Elabscience, Houston, TX, USA), following the manufacturer’s protocols. The analyzed markers included pro-inflammatory cytokines (IL-1β, IL-6, TNF-α), the matrix-degrading enzyme MMP-13, cartilage degradation markers (COMP, CTX-II), and the oxidative stress marker iNOS. Prior to analysis, all samples were stored at −80 °C and thawed only once to avoid freeze–thaw-related degradation.

All ELISA assays were performed using pre-coated sandwich ELISA plates (Elabscience, Houston, TX, USA). The specific catalog numbers were as follows: IL-1β (E-EL-R0012), IL-6 (E-EL-R0015), TNF-α (E-EL-R2856), MMP-13 (E-EL-R0045), CTX-II (E-EL-R2554), COMP (E-EL-R0159), and iNOS (E-EL-R0520). Calibration standards supplied with each kit were prepared according to the manufacturer’s lot-specific instructions, and all assays were conducted using reagents from the same production batch to minimize inter-assay variability.

Plates were blocked with 1% bovine serum albumin (BSA) in PBS for 1 h at room temperature. After washing, samples and standards (100 µL per well) were added and incubated for 90 min at 37 °C. Following a wash step, biotinylated detection antibodies were added and incubated for 60 min at 37 °C. Plates were then washed and incubated with horseradish peroxidase (HRP)-conjugated streptavidin for 30 min at 37 °C. After a final wash, the substrate solution was added for color development, the reaction was stopped, and optical density was measured at 450 nm using a microplate reader (BioTek Instruments, Winooski, VT, USA) [77,78].

All assays were performed in duplicate as technical replicates, and mean values were used for statistical analysis. For cartilage homogenates, concentrations were normalized to total protein content determined by the Bradford assay to account for variability in tissue extraction. Detection ranges and assay sensitivities are summarized in Table 1. Negative control wells (without sample or antigen) and manufacturer-provided positive controls were included on each plate to ensure assay validity and reproducibility. Standard curves were generated in parallel with each run, and only curves with an R^2^ ≥ 0.99 were accepted for quantification.

#### 2.2.4. Total Antioxidant Status (TAS) and Total Oxidative Status (TOS) Assays

Total antioxidant status (TAS) and total oxidative status (TOS) were measured in both synovial fluid and cartilage homogenates according to the automated colorimetric methods originally described by Erel [79,80]. Commercially available kits (Rel Assay Diagnostics, Gaziantep, Turkey) were used following the manufacturer’s instructions.

The TAS assay is based on the ability of antioxidants in the sample to inhibit the formation of the ABTS^+^ radical cation, with results expressed as mmol Trolox equivalent/L [79]. The TOS assay measures the oxidation of ferrous ion to ferric ion by oxidants present in the sample, which then form a colored complex with xylenol orange; results are expressed as μmol H_2_O_2_ equivalent/L [80].

The oxidative stress index (OSI) was calculated as the ratio of TOS (expressed in μmol H_2_O_2_ equivalent/L) to TAS (expressed in mmol Trolox equivalent/L), following appropriate unit conversion (OSI = (TOS/TAS) × 100).

All assays were performed in duplicate, and standard curves were generated for each plate. For cartilage homogenates, TAS and TOS levels were normalized to total protein concentration, as determined by the Bradford assay, to minimize variability.

### 2.3. Statistical Analysis

Statistical analyses were performed using SPSS v27.0 (IBM Corp., Armonk, NY, USA). Distribution of variables was tested with the Shapiro–Wilk test. Data following normal distribution were compared with independent-samples *t*-test; non-normally distributed data were analyzed using the Mann–Whitney U test. Results are presented as mean ± standard deviation (SD) and median (min–max). Sample size (*n* = 10 per group) was determined a priori to ensure sufficient statistical power (β = 0.80, α = 0.05), consistent with previously validated rat osteoarthritis models and standard recommendations for experimental power analysis. Comparable group sizes have been widely used in ACLT-induced OA studies and preclinical NAC investigations in rodents [37,81,82,83,84] and post hoc effect size calculations indicated robust group differences where significance was reached. A two-tailed α of 0.05 was accepted as the threshold for statistical significance.

## 3. Results

### 3.1. Histological Observations

**Figure 1:**
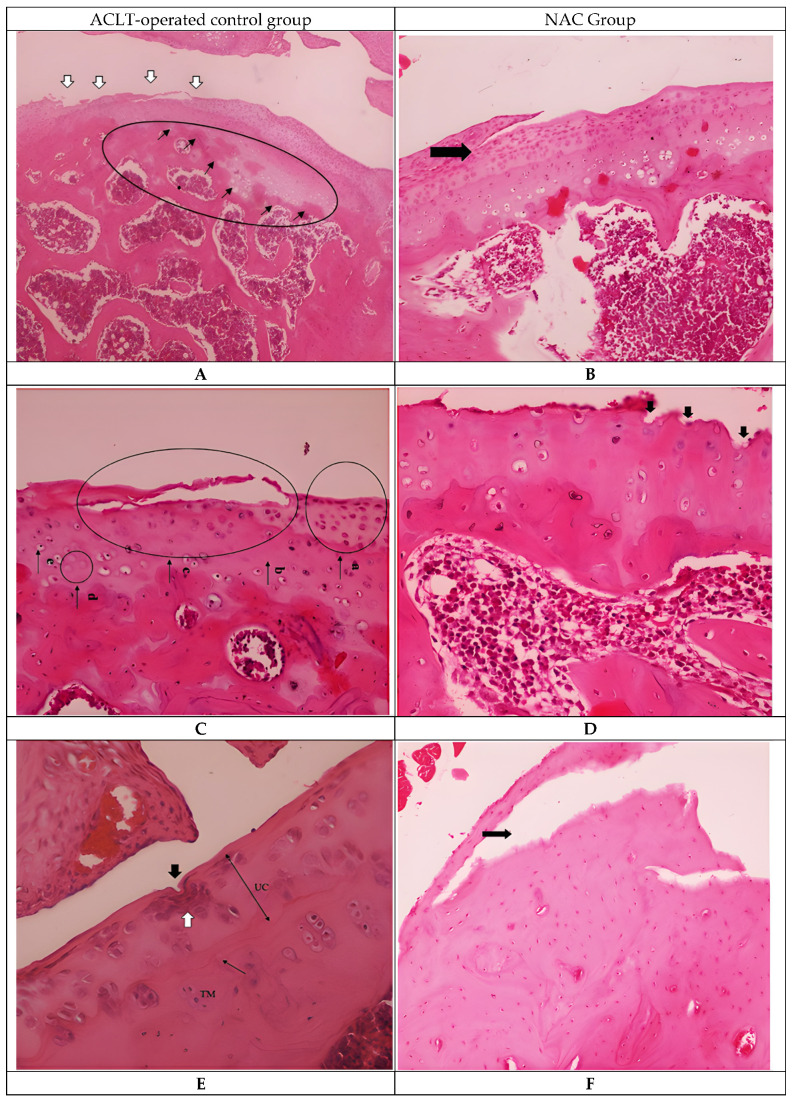

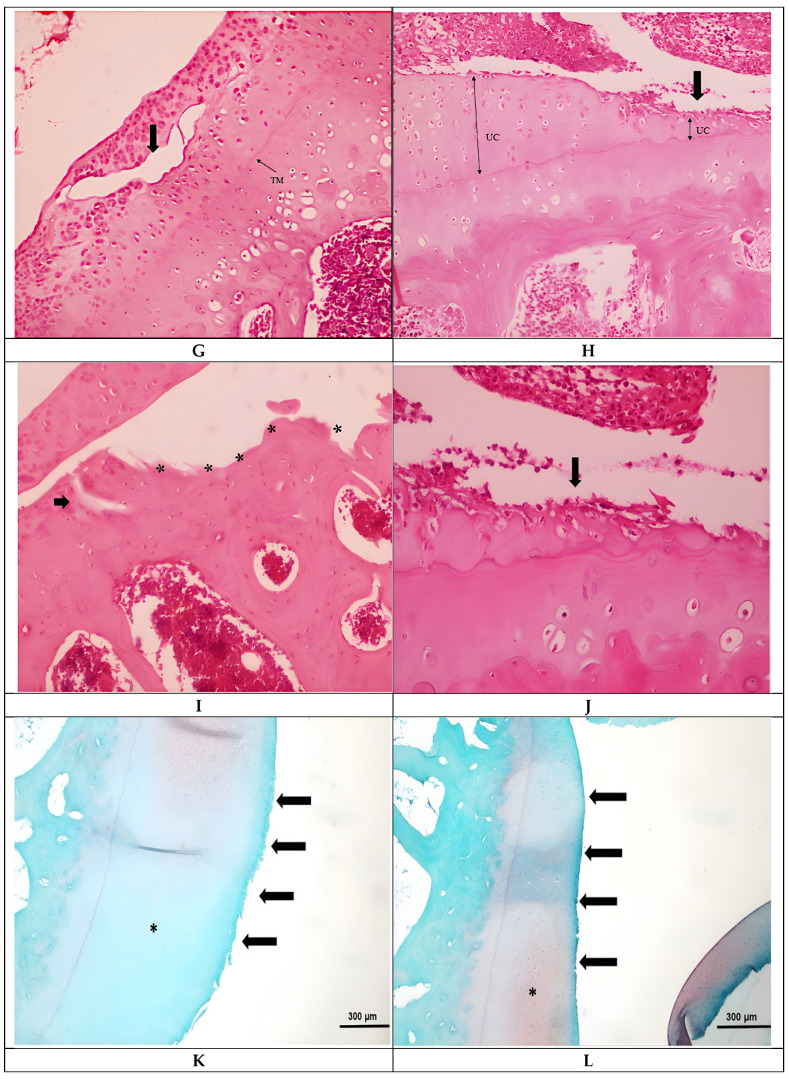
Representative histological sections of rat knee joints from ACLT-operated control and NAC-treated groups stained with hematoxylin–eosin (H&E) and Safranin O/Fast Green. Panels (**A**,**C**,**E**,**G**,**I**,**K**) represent the control group, whereas panels (**B**,**D**,**F**,**H**,**J**,**L**) represent the NAC-treated group. At low magnification, panels (**A**,**B**) demonstrate tidemark irregularities (black arrows) and loss of articular cartilage (white arrows) in the ACLT-operated control group (**A**), and clefts extending to the radial zone (black arrow) in the NAC-treated group (**B**) (H&E, 4× and 20×). At higher magnification, panels (**C**,**D**) show the cartilage–subchondral bone interface, with cartilage tissue (a), bone tissue (b), loss of articular cartilage (c), apoptotic chondrocytes (d), and chondrocytes (e) in the ACLT-operated control group (**C**), while the NAC-treated group (**D**) demonstrates an irregular articular surface, clefts extending to the transitional zone (black arrows), and hypocellularity (H&E, 10× and 40×). Panels (**E**,**F**) demonstrate a cleft extending to the transitional zone (black arrow) accompanied by chondrocyte clustering (white arrow) in the ACLT-operated control group (**E**), and clefts extending to the radial zone (black arrow) with hypocellularity in the NAC-treated group (**F**) (H&E, 40×). In panels (**G**,**H**) clefts extending to the radial zone (black arrow) with cloning of chondrocytes in the transitional and radial layers and marked hypercellularity are observed in the ACLT-operated control group (**G**), while decreased cartilage thickness and partial loss of cartilage (black arrow) are observed in the NAC-treated group (**H**) (H&E, 20×). In advanced degeneration fields, panels (**I**,**J**) demonstrate a cleft extending to the radial zone (black arrow), tidemark irregularity, and loss of articular cartilage (*) in the ACLT-operated control group (**I**), and loss of cartilage (black arrow) with hypocellularity in the transitional and radial zones in the NAC-treated group (**J**) (H&E, 40×). Finally, Safranin O/Fast Green staining in panels (**K**,**L**) demonstrates regions marked with an asterisk (*) showing reduced red staining, indicative of proteoglycan depletion, while black arrows denote areas of articular surface erosion in both groups (×20; scale bar = 100 µm). TM: tidemark; UC: uncalcified cartilage.

Histopathological evaluation of the control and NAC-treated specimens revealed marked degenerative changes in articular cartilage (Figure 1). In the ACLT-operated control group, irregularities of the tidemark and loss of surface cartilage integrity were observed at low magnification (Figure 1A). At higher magnification, extensive matrix damage accompanied by numerous apoptotic chondrocytes and disruption of the cartilage–subchondral bone interface were evident (Figure 1C). Fissures extended into the transitional zone, where clustering of chondrocytes was observed (Figure 1E). In deeper regions, including the radial zone, clefts progressed further, and chondrocyte cloning was noted in both the transitional and radial layers, accompanied by hypercellularity (Figure 1G). Advanced cartilage degeneration was characterized by radial clefts, tidemark distortion, and breakdown of the normal cartilage architecture (Figure 1I). Safranin O/Fast Green staining demonstrated a pronounced reduction in proteoglycan content, evidenced by diminished red staining, along with focal areas of articular surface erosion (Figure 1K).

Similarly, the NAC-treated group exhibited degenerative alterations in articular cartilage (Figure 1). Clefts extending into the radial zone were observed, together with surface irregularities and fissures reaching the transitional zone, accompanied by hypocellularity (Figure 1B,D). Additional clefts involving the radial zone were associated with pronounced hypocellularity (Figure 1F). Decreased cartilage thickness and partial loss of cartilage were evident in deeper layers (Figure 1H). Hypocellularity and cartilage loss persisted in both the transitional and radial zones (Figure 1J). Safranin O/Fast Green staining revealed persistent reduction in proteoglycan content, as indicated by diminished red staining, along with focal articular surface erosion (Figure 1L).

**Figure 2:**
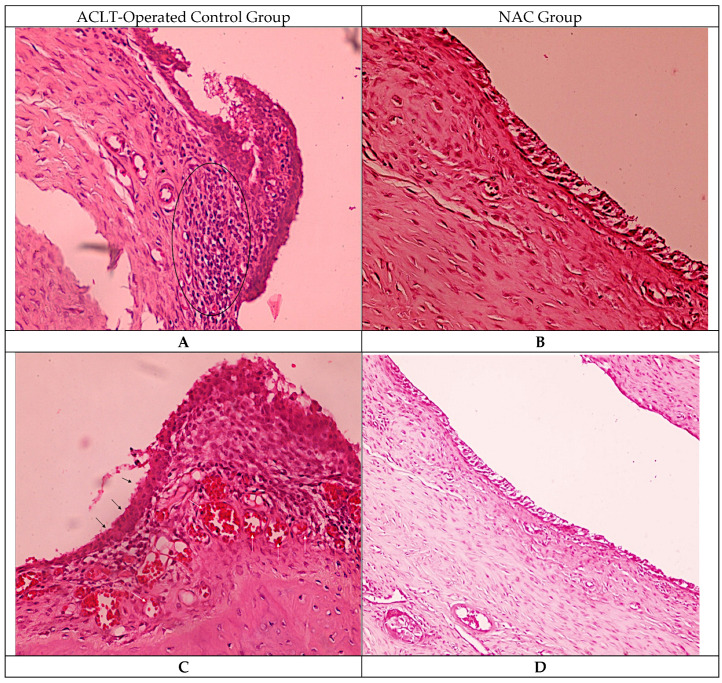
Histological evaluation of synovial tissue Representative hematoxylin–eosin (H&E)–stained sections of synovial tissue obtained from rat knee joints following anterior cruciate ligament transection (ACLT). Panels (**A**–**C**) are shown at 40× magnification, while panel (**D**) is shown at 20× magnification. Panels (**A**,**C**) represent the ACLT-operated control group, showing dense inflammatory cell infiltration within the synovial stroma (elliptical markings in panel (**A**)), synovial lining hyperplasia (black arrows in panel (**C**)), and congested synovial blood vessels (white arrows in panel (**C**)). Panels (**B**,**D**) represent the N-acetylcysteine (NAC)–treated group and demonstrate a relatively thinner synovial lining, reduced stromal cellularity, and less prominent vascular congestion, consistent with attenuation of synovial inflammatory features following NAC administration.

Histological examination of synovial tissue demonstrated distinct morphological features in both groups. In the ACLT-operated control group, synovial sections showed dense inflammatory cell infiltration within the synovial stroma, accompanied by thickening of the synovial lining layer. Areas of synovial lining hyperplasia and marked vascular congestion were frequently observed, with expanded stromal cellularity throughout the synovial membrane. In the NAC-treated group, synovial sections displayed a thinner synovial lining with lower stromal cellular density. Inflammatory cell infiltration appeared less extensive, and vascular structures showed reduced congestion compared with the control specimens. These histological features were consistently observed across examined sections (Figure 2).

Table 2 provides a detailed comparison of the histopathological parameters assessed using the Mankin scoring system for cartilage integrity between the ACLT-operated control group and NAC groups. The parameters include:

Structure: Examines the integrity of cartilage, with scores indicating varying levels of structural damage. The NAC group showed no significant differences compared to the Control group (*p* > 0.05).

Cellularity: Evaluates changes in the number and morphology of chondrocytes. Both groups demonstrated similar cellularity scores, with no statistically significant differences (*p* > 0.05).

Safronin-O Staining: Indicates proteoglycan content in cartilage. The staining scores were comparable between the groups, with no significant reduction observed in the NAC group (*p* > 0.05).

Tide Mark Integrity: Assesses changes at the cartilage-bone interface. The NAC group showed no significant differences in tide mark scores compared to the ACLT-operated control group (*p* > 0.05).

Total Score: Represents the overall assessment of cartilage damage. No statistically significant differences in the total Mankin score were observed between the ACLT-operated control and NAC groups (*p* > 0.05).

### 3.2. Synovial Fluid Biomarker Profiles

**Figure 3:**
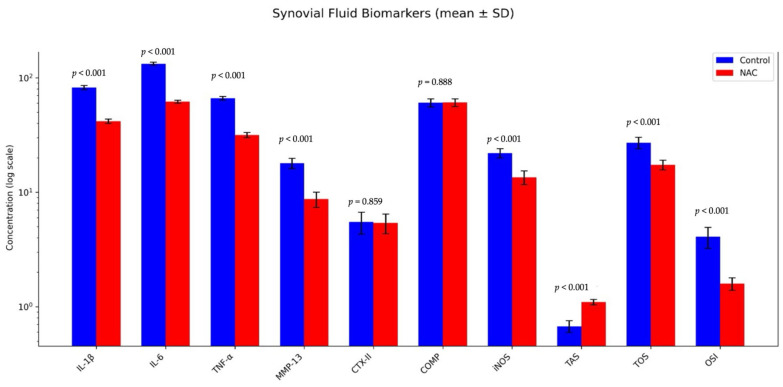
Synovial fluid biomarker profiles. Bar graphs represent synovial fluid concentrations of inflammatory cytokines (IL-1β, IL-6, TNF-α), matrix-degrading enzyme (MMP-13), cartilage degradation markers (CTX-II, COMP), oxidative stress marker (iNOS), and redox balance indices (TAS, TOS, OSI) in ACLT-operated control and NAC-treated groups. Data are presented as mean ± SD on a logarithmic scale. NAC treatment significantly reduced IL-1β, IL-6, TNF-α, MMP-13, iNOS, TOS, and OSI levels, while significantly increasing TAS (all *p* < 0.001). No significant differences were observed for CTX-II or COMP between groups. Statistical comparisons were performed between groups for each parameter.

NAC treatment significantly reduced IL-1, IL-6, TNF-α, MMP-13, and iNOS levels in the synovial fluid compared to the ACLT-operated control group (*p* < 0.05). However, no significant changes were observed for CTX-II and COMP levels (*p* > 0.05). In addition, oxidative stress indices showed marked improvements in the NAC group. TAS levels were significantly increased, indicating enhanced antioxidant capacity, whereas TOS levels were significantly reduced, reflecting decreased oxidative burden (both *p* < 0.001). Consequently, the OSI value—a composite marker of redox balance—was significantly lower in the NAC group compared to controls (*p* < 0.001), confirming that NAC effectively attenuated oxidative stress in the synovial fluid. (Table 3, Figure 3).

### 3.3. Cartilage Biochemical Profiles

**Figure 4:**
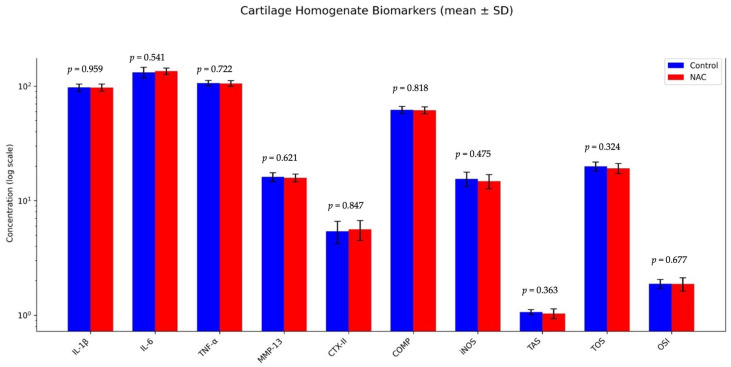
Cartilage homogenate biomarker profiles. Bar graphs depict concentrations of inflammatory cytokines (IL-1β, IL-6, TNF-α), matrix-degrading enzyme (MMP-13), cartilage degradation markers (CTX-II, COMP), oxidative stress marker (iNOS), and redox balance indices (TAS, TOS, OSI) measured in cartilage homogenates obtained from ACLT-operated control and NAC-treated groups. Data are presented as mean ± SD on a logarithmic scale. No statistically significant differences were observed between groups for any analyzed parameter (all *p* > 0.05), indicating that intra-articular NAC administration did not alter inflammatory, oxidative stress-related, or matrix degradation markers within cartilage tissue.

No significant differences were observed between the ACLT-operated control and NAC groups for any markers analyzed in the cartilage homogenate, including IL-1, IL-6, TNF-α, MMP-13, CTX-II, COMP, and iNOS (*p* > 0.05). Similarly, oxidative stress indices (TAS, TOS, OSI) showed no significant changes between groups, indicating that NAC did not alter the redox balance within cartilage tissue (*p* > 0.05) (Table 4, Figure 4).

**Figure 5:**
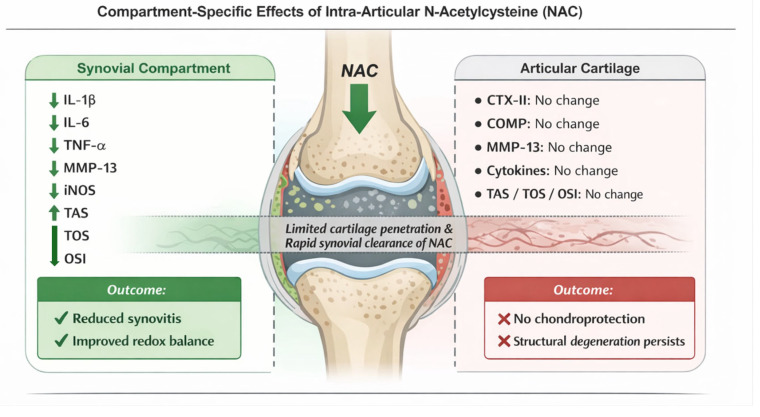
Compartment-specific effects of intra-articular N-acetylcysteine (NAC) on synovial inflammation and cartilage metabolism.

Figure 5 illustrates the compartment-specific effects of NAC on different components of the knee joint. Synovial concentrations of IL-1β, IL-6, TNF-α, and iNOS were significantly reduced following intra-articular NAC administration. Oxidative stress analysis demonstrated a significant decrease in total oxidant status (TOS) and oxidative stress index (OSI), along with a significant increase in total antioxidant status (TAS) in the synovial compartment. In contrast, no statistically significant differences were observed in cartilage-related biomarkers, including CTX-II, COMP, MMP-13, cytokine levels, or oxidative stress parameters between the groups.

## 4. Discussion

Our study demonstrated that N-acetylcysteine (NAC) effectively reduced pro-inflammatory cytokines (IL-1, IL-6, TNF-α), matrix-degrading enzymes (MMP-13), and the oxidative stress marker iNOS in the synovial fluid, highlighting its potent anti-inflammatory and antioxidative effects in the joint microenvironment. Moreover, NAC significantly improved the global redox profile in synovial fluid, as reflected by increased TAS and reduced TOS and OSI levels. These findings confirm that NAC exerts a measurable biological impact within the synovial compartment. These biochemical findings were further supported by qualitative synovial histological observations. Representative synovial sections demonstrated differences in synovial lining thickness, stromal cellularity, inflammatory cell infiltration, and vascular congestion between the control and NAC-treated groups, providing tissue-level morphological context for the observed reductions in synovial inflammatory mediators.

In contrast, NAC did not significantly influence cartilage degradation markers (CTX-II, COMP), nor did it improve histological parameters such as cartilage structure, cellularity, and proteoglycan content. Histopathological analysis revealed clefts extending into the transitional and radial zones, hypocellularity, and cartilage thinning, which remained prominent in the NAC group and were comparable to controls. Similarly, cartilage homogenate analyses showed no significant changes in pro-inflammatory cytokines, oxidative stress indices (TAS, TOS, OSI), or matrix degradation markers. Together, these findings indicate that NAC’s therapeutic actions were compartment-specific—attenuating synovial inflammation and oxidative stress—yet without measurable chondroprotective effects on cartilage tissue.

Our findings partially align with prior research on NAC in osteoarthritis. Özcamlıbel et al. [85] reported that NAC significantly reduced oxidative stress (TOS) and degradation markers (CTX-II, MMP-3) in OA patients, supporting NAC’s antioxidant potential. However, their study involved only 10 patients, lacked stratification by Kellgren–Lawrence severity or effusion status, and did not evaluate cartilage preservation, limiting mechanistic interpretation. Kaneko et al. [37] found NAC reduced MMP-13, preserved type II collagen, and improved Mankin scores in a mechanical stress-induced OA rat model. Yet, their systemic oral administration differs from our intra-articular design, and they did not biochemically separate synovial and cartilage compartments. Nakagawa et al. [36] further demonstrated that NAC restored glutathione levels and suppressed ROS-induced apoptosis in vitro and reduced oxidative stress in ACLT rat models, aligning with our synovial oxidative stress results. However, their use of rabbit cartilage in vitro and rat models in vivo, without cartilage-specific biochemical markers, limits direct translation. More recently, Özdemir et al. [86] reported that intra-articular NAC improved histological parameters in a rat osteochondral defect model; however, their analyses were limited to morphology and did not include biochemical markers or compartment-specific evaluation, restricting mechanistic insight.

Other studies highlight the complexity of NAC’s effects. Dycus et al. [87] showed NAC restored superoxide dismutase (SOD), increased glutathione (GSH), and reduced prostaglandin E2 (PGE2) in canine chondrocytes exposed to oxidative stress. While consistent with our observation of reduced oxidative markers, their in vitro system cannot replicate mechanical stress or subchondral bone remodeling. Riegger et al. [38] reported NAC reduced MMP-13 and preserved proteoglycan content in a trauma-induced PTOA model, suggesting protection in acute phases. However, their short-term trauma setting contrasts with our chronic ACLT model, which mimics long-term instability and degeneration. Moreover, they did not assess synovial cytokines such as IL-1, IL-6, or TNF-α. Our findings are consistent with those of Roman-Blas et al. [39], who showed that NAC suppressed NF-κB, COX-2, and MMP-13 in osteoarthritic synoviocytes but failed to inhibit catabolic mediators in IL-1β-stimulated chondrocytes, even enhancing MMP-13 expression. While their observations were limited to in vitro conditions, our in vivo ACLT model corroborates this compartment-specific effect, demonstrating that NAC effectively reduces synovial inflammation but does not prevent cartilage degeneration in a living system that reproduces the multifactorial OA microenvironment. Finally, Yeh et al. [40], in a large retrospective cohort, linked long-term oral NAC to increased OA risk, possibly via altered p53 signaling in chondrocytes. Their absence of biomarker data prevents mechanistic confirmation, but their results parallel our observation that NAC did not protect cartilage despite reducing synovial inflammation.

Collectively, these studies emphasize the heterogeneous and context-dependent effects of NAC in OA. Our study adds novelty by performing a compartment-specific biochemical assessment of both synovial fluid and cartilage homogenates, together with oxidative stress indices (TAS, TOS, OSI). This integrated approach demonstrates that NAC consistently attenuates inflammation and oxidative stress within the synovial environment but does not modify cartilage catabolism in a chronic ACLT setting. By aligning biochemical and histological readouts, our findings extend previous reports and help explain why NAC appears effective in acute or in vitro conditions, yet fails to provide durable chondroprotection in progressive post-traumatic OA.

Several mechanistic factors may explain why intra-articular NAC successfully attenuated synovial inflammation and oxidative stress but failed to preserve cartilage integrity in our ACLT model. First, tissue permeability and pharmacokinetics are crucial. The synovium is vascularized, metabolically active, and directly exposed to the joint milieu, allowing NAC to diffuse and exert its antioxidant effects. In contrast, articular cartilage is avascular, embedded within a dense proteoglycan- and collagen-rich extracellular matrix that imposes steric and electrostatic barriers to the penetration of small hydrophilic molecules such as NAC. Consequently, effective drug concentrations may not be reached in deeper cartilage zones. This challenge is compounded by the rapid clearance of small molecules from the synovial cavity—often within hours—limiting sustained intra-cartilage exposure [88,89,90]. Second, redox biology and oxygen tension differ sharply between synovium and cartilage. Synoviocytes exist in a relatively oxygen-rich environment (>12% O_2_) and are highly sensitive to ROS-driven NF-κB signaling, which NAC effectively suppresses. Chondrocytes, however, inhabit a hypoxic niche (3–9% O_2_) and rely on HIF-mediated adaptations that alter ROS handling. In this setting, NAC has paradoxically been shown to enhance MMP-13 expression under IL-1β stimulation, reflecting context-dependent crosstalk between HIF-1α and NF-κB pathways [39,91,92,93]. Such cell-specific differences likely explain the observed dissociation between synovial and cartilage responses. Third, crosstalk between synovium, cartilage, and subchondral bone creates reinforcing feedback loops. Synovitis drives the release of cytokines and proteases such as IL-1, IL-6, TNF-α, and MMP-13, which promote cartilage breakdown. Cartilage-derived degradation products, in turn, act as damage-associated molecular patterns (DAMPs), perpetuating synovial inflammation. Meanwhile, tidemark disruption enables vascular invasion from subchondral bone, introducing growth factors such as TGF-β that further sustain catabolism [94,95,96]. Although NAC suppressed synovial mediators, persistent cartilage degradation may have continued to fuel these joint-wide feedback cycles. Fourth, cellular senescence and non-ROS-mediated mechanisms further limit NAC’s efficacy. Senescent chondrocytes accumulate in OA and adopt a SASP phenotype, releasing IL-6, TNF-α, MMPs, and other catabolic mediators. These processes are closely linked to mitochondrial dysfunction, DNA damage, and epigenetic alterations, which are not reversed by simple ROS scavenging. The concept of “inflammosenescence” describes a self-sustaining loop between cellular aging and chronic inflammation [97], suggesting that while NAC may neutralize oxidative stress, it cannot halt senescence-driven catabolism in cartilage. Finally, subchondral bone remodeling in the ACLT model represents another driver of ongoing degeneration. Abnormal loading induces bone turnover, vascular invasion, and release of osteogenic factors that propagate cartilage loss [98,99,100]. Since NAC does not directly influence these bone-derived pathways, its chondroprotective potential remains limited in the context of mechanically driven, chronic OA. Importantly, the lack of changes in cartilage oxidative stress indices (TAS, TOS, and OSI) should not be interpreted as an absence of antioxidant activity, but rather as a consequence of limited drug penetration and compartment-specific redox regulation. Increasing NAC dose or injection frequency alone is unlikely to overcome these barriers, as rapid synovial clearance and restricted diffusion into the dense cartilage matrix constrain sustained intra-cartilage exposure. Therefore, the insensitivity of cartilage redox indices most likely reflects pharmacokinetic limitations rather than insufficient dosing.

Taken together, these mechanistic considerations provide the framework for interpreting our study’s strengths and limitations. A key strength was the use of a robust ACLT-induced OA model, which reliably reproduces chronic mechanical instability, synovial inflammation, and progressive cartilage degeneration, thereby providing a clinically relevant post-traumatic OA setting. Importantly, we performed parallel analyses of synovial fluid and cartilage homogenates in conjunction with histological evaluation. This integrated approach enabled a broader assessment of NAC’s effects than studies limited to in vitro experiments or systemic administration, and allowed simultaneous evaluation of both symptomatic and structural aspects of OA pathology.

Nonetheless, several limitations must be acknowledged. The ACLT model, while widely employed in preclinical research, primarily reflects rapidly progressive, post-traumatic OA [60,62,63] and does not fully capture the multifactorial, slowly evolving features of idiopathic or age-related disease, where metabolic, hormonal, and low-grade inflammatory processes play critical roles [52,101]. Moreover, the accelerated degeneration observed in ACLT may exaggerate catabolic pathways while underrepresenting low-grade inflammation and chondrocyte senescence, processes central to human OA [102,103,104]. Thus, although ACLT is well suited for proof-of-concept studies, extrapolation of its findings to all OA phenotypes should be approached with caution.

In addition, cartilage degeneration in the ACLT model is known to be spatially heterogeneous, with the medial compartment being preferentially affected due to altered joint biomechanics. Although Mankin scoring in the present study was intentionally restricted to the medial tibiofemoral compartment to capture consistent and clinically relevant degeneration, this region-specific approach may underestimate pathological changes occurring in other joint compartments. Future studies incorporating multi-compartmental scoring strategies or whole-joint quantitative histomorphometric analyses would provide a more comprehensive assessment of regional cartilage pathology.

More specifically, the pathophysiological disparities between the ACLT model and human OA warrant careful consideration. Our model recapitulates the acute mechanical overload and instability seen in post-traumatic OA, characterized by rapid synovitis and cartilage breakdown driven by biomechanical stress [60,62]. In contrast, human idiopathic OA typically follows a slow, progressive course over decades, where low-grade inflammation, chondrocyte senescence, and metabolic dysregulation (e.g., related to aging, obesity, or diabetes) play predominant roles [101,102]. Furthermore, the ACLT model does not fully encompass the hormonal influences (e.g., estrogen deficiency in postmenopausal OA) or the systemic low-grade inflammatory milieu often present in human disease [52,104]. This discrepancy may explain why an antioxidant/anti-inflammatory agent like NAC showed compartment-specific efficacy in our model but failed to halt the structurally driven cartilage degeneration, a process potentially more dominant in this mechanical injury setting than in early metabolic or age-related OA.

These mechanistic differences introduce potential translational biases. First, a temporal bias exists: the 6-week accelerated degeneration in rats may not reflect the chronicity of human OA, possibly overlooking any slow-acting, long-term protective effects of NAC. Second, there is an etiological bias: our findings are most directly applicable to post-traumatic or instability-driven OA, whereas the therapeutic window for NAC might differ in early idiopathic OA with a stronger inflammatory component [105,106]. Third, a pharmacokinetic bias must be considered; the intra-articular dosing regimen optimized for rats may not directly translate to effective human joint pharmacokinetics or patient adherence. Therefore, while our data robustly indicate that NAC alone is insufficient to modify structural progression in a setting of established mechanical instability, they do not preclude its potential utility as a symptom-modifying agent in human OA subsets with significant synovitis. Future clinical translation should involve patient stratification based on OA phenotype (e.g., inflammatory vs. degenerative) [107] and may consider NAC as part of a combination therapy targeting both synovial inflammation and cartilage/bone remodeling.

These pharmacokinetic limitations highlight the need for advanced intra-articular delivery strategies to enhance drug retention and tissue-specific bioavailability. Small, hydrophilic molecules such as N-acetylcysteine are rapidly cleared from the synovial cavity, limiting sustained intra-articular exposure and restricting diffusion into the dense extracellular matrix of articular cartilage. Emerging delivery platforms, including hydrogel-based carriers, nanoparticle systems, and conjugated drug formulations, have demonstrated prolonged intra-articular residence time and improved cartilage penetration in experimental osteoarthritis models. Incorporation of such approaches may overcome rapid clearance and enhance the therapeutic potential of N-acetylcysteine by enabling sustained, localized release within the joint environment. Future studies should therefore explore biomaterial-based NAC delivery systems to optimize pharmacokinetics and determine whether improved joint retention translates into structural cartilage protection.

Although subchondral bone remodeling is a well-established driver of cartilage degeneration in the ACLT model, the present study did not include a direct assessment of bone mineral density (BMD) or trabecular microarchitecture using micro-computed tomography (micro-CT). Consequently, we cannot exclude the possibility that persistent aberrant subchondral bone remodeling—potentially unaffected by intra-articular NAC—contributed to the continued cartilage degeneration observed in this model. Future studies integrating longitudinal micro-CT analysis with biochemical and histological assessments will be essential to elucidate the temporal and mechanistic interplay between subchondral bone remodeling and cartilage degeneration in post-traumatic osteoarthritis.

An additional limitation of the present histological assessment is the absence of a naïve (non-operated) cartilage control for Safranin-O staining. While Safranin-O is widely used to assess proteoglycan content, its interpretation is inherently relative. In ACLT-induced rat models, complete loss of Safranin-O staining is uncommon, and reductions typically reflect partial depletion rather than total proteoglycan loss. Accordingly, the diminished Safranin-O staining observed in both control and NAC-treated groups in our study should be interpreted as a relative decrease compared with expected normal cartilage staining patterns, rather than absolute depletion. The inclusion of naïve cartilage controls in future studies would further strengthen quantitative and qualitative interpretation of proteoglycan changes.

Other methodological constraints should also be noted. We restricted evaluation to a single endpoint, which may have overlooked dynamic temporal effects, and did not directly measure NAC penetration into cartilage. Our inflammatory panel focused only on canonical cytokines (IL-1β, IL-6, TNF-α), excluding other mediators such as CRP or IL-8 that may contribute to low-grade inflammation. Furthermore, while our dosing regimen (5 mg/50 µL administered intra-articularly once weekly for 3 weeks) was selected based on prior experimental studies demonstrating local efficacy in rodent osteoarthritis models [36,38], it represents a single, fixed-dose approach. Consequently, the optimal therapeutic dose of NAC cannot be determined from the present data, and it remains unclear whether alternative dosing strategies—such as higher or lower doses, increased administration frequency, or prolonged treatment duration—might yield enhanced or sustained cartilage-protective effects. In addition, the accelerated disease course inherent to the ACLT model may impose pharmacokinetic and pharmacodynamic demands that differ substantially from the slowly progressive nature of human osteoarthritis. Therefore, the translational applicability of the current dosing schedule should be interpreted with caution. Future preclinical studies should systematically investigate dose–response relationships and extended treatment protocols, as well as evaluate the long-term safety profile of intra-articular NAC, including potential local joint tissue toxicity, to better inform the rational design of clinical trials and optimize therapeutic regimens. Although synovial fluid collection was standardized with a perfusion technique, total protein normalization was not performed, which could have further minimized variability. Although qualitative histological evaluation of the synovial membrane was performed, a formal synovitis scoring system was not applied. Therefore, direct quantitative correlation between synovial histopathological changes and biochemical inflammatory markers could not be established. Tissue-level mechanistic analyses such as immunohistochemistry, PCR, or Western blotting were also absent, and H&E assessment was limited to the Mankin scoring system without quantitative or cell-specific analyses. Finally, inherent interspecies differences between rats and humans must be considered when interpreting the translational relevance of our results.

The present study underscores that intra-articular NAC exerts clear anti-inflammatory and antioxidative effects within the synovial compartment but fails to provide measurable chondroprotection in cartilage tissue. These findings carry important clinical implications. Since synovitis and oxidative stress are central drivers of pain and functional impairment in OA, NAC may hold therapeutic value in patients with inflammation-dominant phenotypes, acting as a symptom-modifying agent. However, the absence of structural cartilage preservation suggests that NAC alone is unlikely to alter long-term disease progression. This highlights the need to investigate NAC in combination with agents that directly target cartilage and subchondral bone, thereby addressing the multifactorial pathophysiology of OA.

Future research should expand biomarker profiling to include senescence-associated mediators, HIF-related pathways, and broader antioxidant indices such as SOD, GSH, and NADPH. Incorporation of tissue-level techniques (immunohistochemistry, PCR, Western blot) will be critical to validate tissue-specific effects and assess intra-cartilage penetration. Advances in intra-articular delivery strategies—such as nanoparticles, hydrogels, and conjugated formulations—may also enhance NAC’s bioavailability and retention, overcoming pharmacokinetic limitations. Finally, comparative studies across idiopathic, metabolic, and post-traumatic OA models, followed by carefully stratified clinical trials, are warranted to clarify whether NAC can serve as a context-dependent adjunct in precision medicine approaches to OA management.

## 5. Conclusions

Intra-articular NAC significantly attenuated synovial inflammation and oxidative stress in our ACLT-induced osteoarthritis model, but it failed to confer measurable chondroprotection, as cartilage degeneration persisted despite synovial improvement. These results suggest that NAC may act as a valuable symptom-modifying adjunct in inflammation-dominant OA phenotypes, yet its inability to alter structural progression highlights the necessity of combination approaches targeting cartilage and subchondral bone. Future preclinical and translational studies, particularly those employing advanced delivery systems and phenotype-stratified designs, are warranted to determine whether NAC can evolve from a symptomatic agent into a true disease-modifying therapy.

## Figures and Tables

**Table 1:** Characteristics of ELISA kits used in the study.

Marker	Sensitivity	Detection Range
IL-1β	18.75 pg/mL	31.25–2000 pg/mL
IL-6	7.5 pg/mL	12.5–800 pg/mL
TNF-α	9.38 pg/mL	15.63–1000 pg/mL
MMP-13	0.19 ng/mL	0.31–20 ng/mL
CTX-II	0.1 ng/mL	0.16–10 ng/mL
COMP	0.94 ng/mL	1.56–100 ng/mL
iNOS	0.19 ng/mL	0.31–20 ng/mL

**Table 2:** Histopathological evaluation of cartilage integrity in ACLT-operated control and NAC groups.

	ACLT-Operated Control Group	NAC Group	*p* Value	Effect Size
Structure	5.0 (4–6) (5.0 ± 0.82)	5.0 (4–6) (4.9 ± 0.74)	0.776 *	0.06
Cellularity	2.0 (1–3) (2.3 ± 0.67)	2.0 (2–3) (2.1 ± 0.32)	0.322 *	0.22
Safronin-O staining	3.0 (2–4) (3.0 ± 0.82)	3.0 (2–4) (2.7 ± 0.67)	0.392 *	0.19
Tidemark	1.0 (0–1) (0.8 ± 0.42)	1.0 (0–1) (0.6 ± 0.52)	0.342 *	0.21
Total	11.0 (8–14) (11.1 ± 1.97)	11.0 (8–13) (10.3 ± 1.57)	0.328 **	0.45

Data are presented as median (min-max) (mean ± SD). Group comparisons were performed using independent samples *t*-tests (**) for normally distributed variables and Mann–Whitney U tests (*) for non-normally distributed variables based on Shapiro–Wilk test.

**Table 3:** Synovial fluid concentrations of biochemical markers in ACLT-operated control and NAC groups.

Synovial Fluid	ACLT-Operated Control Group	NAC Group	*p* Value	Effect Size
IL-1 (pg/mL)	81.7 (77.5–88.1) (82.2 ± 3.50)	41.9 (38.7–44.1) (41.7 ± 1.92)	<0.001 *	0.85
IL-6 (pg/mL)	131.8 (127.5–140.1) (132.6 ± 3.97)	61.8 (58.7–64.3) (61.7 ± 1.86)	<0.001 *	0.85
TNF-α (pg/mL)	65.7 (62.5–70.1) (66.2 ± 2.55)	31.2 (29.3–34.1) (31.6 ± 1.70)	<0.001 *	0.85
MMP-13 (ng/mL)	17.8 (15.2–21.4) (17.9 ± 1.87)	8.7 (6.6–10.9) (8.7 ± 1.31)	<0.001 *	0.85
CTX-II (ng/mL)	5.5 (3.4–7.7) (5.5 ± 1.20)	5.4 (3.9–7.0) (5.4 ± 1.05)	0.859 **	0.08
COMP (ng/mL)	60.0 (52.8–68.3) (60.5 ± 5.01)	60.8 (54.7–67.8) (60.8 ± 4.67)	0.888 **	0.06
iNOS (ng/mL)	22.1 (18.5–24.9) (22.0 ± 2.06)	13.5 (11.1–16.5) (13.5 ± 1.84)	<0.001 **	4.36
TAS (mmol Trolox/L)	0.674 (0.54–0.79) (0.674 ± 0.079)	1.098 (1.01–1.20) (1.098 ± 0.060)	<0.001 *	0.85
TOS (µmol H_2_O_2_/L)	27.03 (23.1–32.6) (27.03 ± 3.12)	17.37 (15.1–19.7) (17.37 ± 1.70)	<0.001 *	0.85
OSI (arbitrary unit)	4.08 (3.31–6.04) (4.08 ± 0.85)	1.59 (1.34–1.88) (1.59 ± 0.20)	<0.001 *	0.85

Data are presented as median (min-max) (mean ± SD). Group comparisons were performed using independent samples *t*-tests (**) for normally distributed variables and Mann–Whitney U tests (*) for non-normally distributed variables based on Shapiro–Wilk test.

**Table 4:** Concentrations of biochemical markers in cartilage homogenates of ACLT-operated control group and NAC groups.

Cartilage Homogenate	ACLT-Operated Control Group	NAC Group	*p* Value	Effect Size
IL-1 (pg/mL)	96.9 (88.5–110.1) (97.2 ± 7.23)	96.7 (87.3–108.9) (97.1 ± 6.97)	0.959 **	0.02
IL-6 (pg/mL)	136.4 (102.8–150.2) (131.9 ± 13.9)	136.8 (119.6–148.9) (135.2 ± 8.85)	0.541 **	0.28
TNF-α (pg/mL)	106.4 (98.3–115.7) (106.4 ± 5.74)	105.6 (97.5–116.2) (105.5 ± 5.91)	0.722 **	0.16
MMP-13 (ng/mL)	16.2 (13.9–18.5) (16.1 ± 1.42)	15.9 (14.2–18.2) (15.8 ± 1.26)	0.621 **	0.23
CTX-II (ng/mL)	5.4 (4.1–7.4) (5.4 ± 1.19)	5.6 (3.9–7.3) (5.6 ± 1.11)	0.847 **	0.09
COMP (ng/mL)	61.6 (56.3–70.1) (62.0 ± 4.55)	61.3 (55.4–68.4) (61.6 ± 4.35)	0.818 **	0.11
iNOS (ng/mL)	15.4 (12.1–19.0) (15.5 ± 2.21)	14.8 (12.1–18.2) (14.8 ± 2.08)	0.475 **	0.33
TAS (mmol Trolox/L)	1.064 (0.99–1.15) (1.064 ± 0.054)	1.033 (0.92–1.20) (1.033 ± 0.101)	0.363 *	0.20
TOS (µmol H_2_O_2_/L)	19.94 (16.9–22.0) (19.94 ± 1.82)	19.16 (16.7–22.1) (19.16 ± 1.98)	0.324 *	0.22
OSI (arbitrary unit)	1.88 (1.61–2.14) (1.88 ± 0.17)	1.87 (1.39–2.13) (1.87 ± 0.25)	0.677 *	0.09

Data are presented as median (min-max) (mean ± SD). Group comparisons were performed using independent samples *t*-tests (**) for normally distributed variables and Mann–Whitney U tests (*) for non-normally distributed variables based on Shapiro–Wilk test.

## Data Availability

The data presented in this study are available on request from the corresponding author.

## Abbreviations

The following abbreviations are used in this manuscript:

OA	Osteoarthritis
KOA	Knee Osteoarthritis
ACLT	Anterior Cruciate Ligament Transection
NAC	N-acetylcysteine
IA	Intra-articular
PBS	Phosphate-Buffered Saline
H&E	Hematoxylin and Eosin
ROS	Reactive Oxygen Species
NO	Nitric Oxide
iNOS	Inducible Nitric Oxide Synthase
IL	Interleukin
IL-1β	Interleukin-1 beta
IL-6	Interleukin-6
TNF-α	Tumor Necrosis Factor-alpha
MMP-13	Matrix Metalloproteinase-13
COMP	Cartilage Oligomeric Matrix Protein
CTX-II	C-terminal Cross-linked Telopeptides of Type II Collagen
SD	Standard Deviation
ELISA	Enzyme-Linked Immunosorbent Assay
